# The X-ray Structures of Six Octameric RNA Duplexes in the Presence of Different Di- and Trivalent Cations

**DOI:** 10.3390/ijms17070988

**Published:** 2016-06-27

**Authors:** Michelle F. Schaffer, Guanya Peng, Bernhard Spingler, Joachim Schnabl, Meitian Wang, Vincent Olieric, Roland K. O. Sigel

**Affiliations:** 1 Department of Chemistry, University of Zurich, Winterthurerstrasse 190, Zürich CH-8057, Switzerland; michelle.schaffer@chem.uzh.ch (M.F.S.); spingler@chem.uzh.ch (B.S.); joachim@schnabl.ch (J.S.); 2 Swiss Light Source, Paul Scherrer Institute, Villigen CH-5232, Switzerland; guanya.peng24@gmail.com (G.P.); meitian.wang@psi.ch (M.W.); vincent.olieric@psi.ch (V.O.)

**Keywords:** RNA, X-ray crystallography, bioinorganic chemistry, di- and trivalent cations

## Abstract

Due to the polyanionic nature of RNA, the principles of charge neutralization and electrostatic condensation require that cations help to overcome the repulsive forces in order for RNA to adopt a three-dimensional structure. A precise structural knowledge of RNA-metal ion interactions is crucial to understand the mechanism of metal ions in the catalytic or regulatory activity of RNA. We solved the crystal structure of an octameric RNA duplex in the presence of the di- and trivalent metal ions Ca^2+^, Mn^2+^, Co^2+^, Cu^2+^, Sr^2+^, and Tb^3+^. The detailed investigation reveals a unique innersphere interaction to uracil and extends the knowledge of the influence of metal ions for conformational changes in RNA structure. Furthermore, we could demonstrate that an accurate localization of the metal ions in the X-ray structures require the consideration of several crystallographic and geometrical parameters as well as the anomalous difference map.

## 1. Introduction

Metal ions play a crucial role in the folding of RNA and its catalytic mechanism, which are central in RNA biology. Folded RNA is involved in almost every aspect of cellular metabolism, including protein synthesis, RNA splicing, catalysis, and gene regulation [1,2,3,4], which is possible due to the multifunctional nature of RNA [3]. Cations influence the folding pathway by bringing together unfolded molecules, promoting the formation of secondary structures, stabilizing intermediate structures, and by maintaining the final native structure [3,5,6].

Nucleic acids, with their negative charge, are excellent targets for metal ions and metal-containing compounds. Most of the metal ions interact non-covalently with RNA, for example by electrostatic attraction, outersphere binding via hydrogen bonds, π–π interactions between a ligand of the metal complex and the nucleobases, or shape selective binding to the grooves [7,8]. A second type of binding, called innersphere binding, occurs directly between a metal ion and atoms of the RNA [9]. The MINAS (*M*etal *I*ons in *N*ucleic *A*cid*S*) database [10] allows searching for any specific metal binding sites found in nucleic acids and it distinguishes between outer- and innersphere coordination.

There is no ideal spectroscopic or biochemical method that provides in a single experiment details of metal ion occupation sites, specific ligands environment, and structural response of nucleic acids to cations. Biochemical and chemical methods predict metal ion binding sites and their involvement in catalytic activities of RNA, but this requires either RNA modification or cleavage, or the exchange of the natural cofactor by another metal ion [11,12,13]. Nuclear Magnetic Resonance (NMR) indicates metal position by monitoring changes in chemical shifts of different nuclei upon the addition of metal ions and provides information on dynamic ion-RNA interactions in solution [14,15,16]. Another biophysical approach is Electron Paramagnetic Resonance Spectroscopy (EPR); it is often used to obtain information about coordination environments, but limited to spectroscopic active species [17,18,19,20]. Raman spectroscopy has been used to investigate metal ion binding to the nucleic acid backbone [21]. Selective metal ion excitation by different incident synchrotron radiation in X-ray absorption spectroscopy (XAS) helps to identify metal ion occupancies and gives structural information, but it requires that the metal ion is tightly bound [22].

X-ray crystallography is an excellent method to locate metal ions precisely. Nevertheless, small molecules and metal ions are often misinterpreted in macromolecular structures if only parameters as distances, coordination numbers, B-factors, and valence are verified. A recent study reported that around 10% of the metal ion binding sites in macromolecular structures are problematic and over 30% cannot be identified with sufficient evidence. The CheckMyMetal web server [23] uses eight parameters to evaluate the consistency, the valence, and coordination sphere of each metal ion binding sites. However, there are some limitations in using this webserver for π-backbonding, sites with an asymmetrical ligand arrangement, and for metal ions with more than one oxidation state.

In a previous study 13 different metal ions were localized in two 23-nucleotide long subtypes of the RNA dimerization initiation site (DIS) in the human immunodeficiency virus (HIV)-1 RNA genome. Despite similar sequences the two structures showed different metal preferences and binding sites [24]. We extend the knowledge of this study by describing for the first time a Cu^2+^ binding site and an uncommon interaction of most tested metal ions to O4 of uracil in an octameric RNA duplex. From the crystal structure in the presence of Ca^2+^ we could collect a high quality and redundant data set to get sufficient anomalous signal to solve the crystal with native-SAD (single-wavelength anomalous diffraction). Furthermore, five octameric RNA crystal structures were solved with metal ions appearing less relevant in the context of nucleic acid binding, although they are often applied as chemical probes in nucleic acid biochemistry, as crystallization agents, or as Mg^2+^-mimicking atoms. Therefore, it was important to determine the sites of metal ion binding with confidence, which was achieved by collecting sufficient anomalous signals of four metal ions at different wavelengths together with careful inspection of the B-factor, valence bond parameter [25], and metal ligand distance.

## 2. Results

### 2.1. Overall Structure

**Figure 1:**
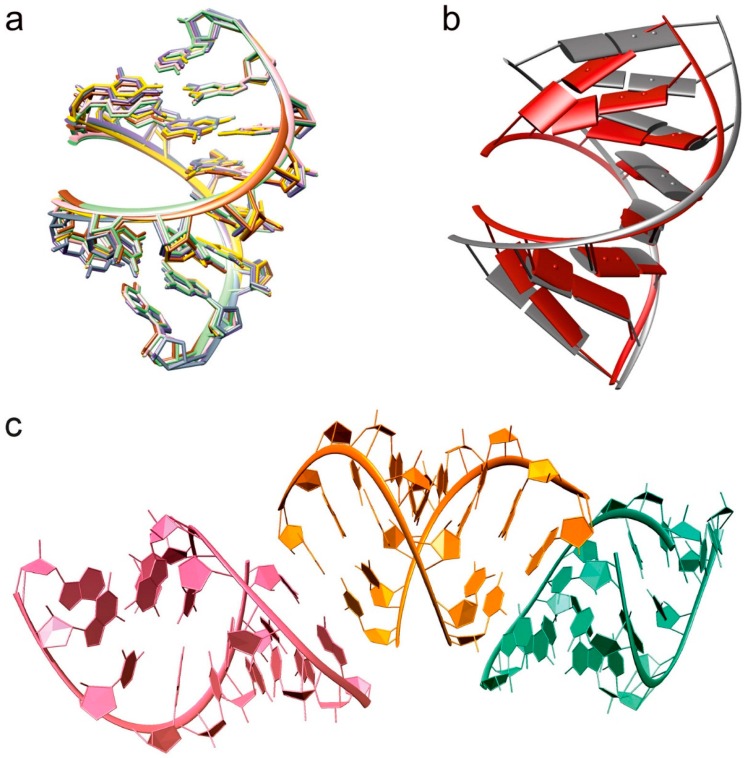
Effects of the nature of the metal ion on RNA structure. (**a**) Overlay of all octameric RNA crystal structures in the presence of Ca^2+^ (**green**), Mn^2+^ (**purple**), Sr^2+^ (**grey**), Tb^3+^ (**yellow**), Cu^2+^ (**brown**), and Co^2+^ (**pink**); (**b**) Calculated, energy-minimized octameric A-RNA duplex (**grey**) in solution compared to the X-ray structure solved in the presence of Ca^2+^ (**red**). The energy-minimized octameric A-RNA duplex was calculated with the program RNAComposer [28]; (**c**) end-on-end stacking of individual octamers (shown in three different colors) in the crystal structure.

In order to investigate the influence of metal ions on RNA structure, six octameric RNA duplex structures were solved in the presence of different metal ions. The RNA forms a continuous helix throughout the lattice by end-to-end stacking of the asymmetric unit (Figure 1), as it is often seen in crystal structures of nucleic acids to maximize the energy of the base-stacking [26]. Despite crystallized structures from solutions containing different metal ions, no particular structural changes were observed of the octameric RNA duplex (Figure 1). The isomorphous structures showed the characteristics of the A-form helix. However, comparison with a calculated “ideal” A-RNA duplex in solution reveals that the RNA duplex in the presence of the metal ions is more compressed and twisted (Figure 1). Quantitative data from the analysis of the three-dimensional structure by using the program 3DNA [27] confirmed these observations. In the presence of metal ions the values for the helical rise and the major groove width of the RNA are smaller, whereas the helical twist is bigger (Table 1). The compaction is particularly visible at the first nucleobases of the strand, which are more out-of-plane twisted to each other than in the “ideal” octameric RNA, and additionally hydrogen bond formation from U1O5′ to OP2 of C2 of the other strand is observed (Figure 1b). In addition, the quantitative analysis reveals that the structures in the presence of Mn^2+^, Sr^2+^, and Tb^3+^ have a slightly bigger major groove width (Table 1). This could be either due to the bigger ionic radius or that they are less effective in compensating the electrostatic repulsion.

### 2.2. Careful Examination of the Tested Metal Ions

The first crystal structure solved by native-SAD [29,30] was co-crystallized with Ca^2+^. Three of the other RNA crystals were grown in the presence of calcium(II) and afterwards soaked in solutions containing either Mn^2+^, Cu^2+^, or Tb^3+^. We measured all solved crystal structures at the corresponding X-ray absorption edge to get a strong anomalous signal. We could thereby unambiguously localize the ions by calculating anomalous difference maps in various resolution ranges. Further we verified that the B-factor of the metal and the environment are in the same range and we considered the distance and coordination to surrounding waters (Table 2).

**Figure 2:**
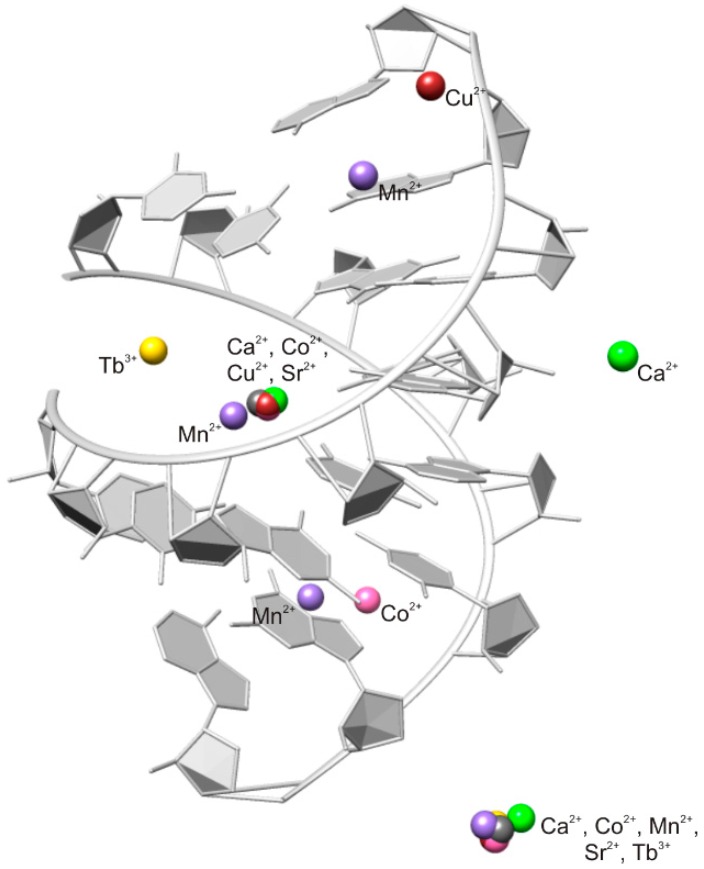
Positions of the metal ions found in different crystal structures of the octameric RNA duplex. The atoms of all RNA strand structures are superimposed, although the RNA duplex shown is the one solved in the presence of Ca^2+^, whereas the other structures are hidden.

**Figure 3:**
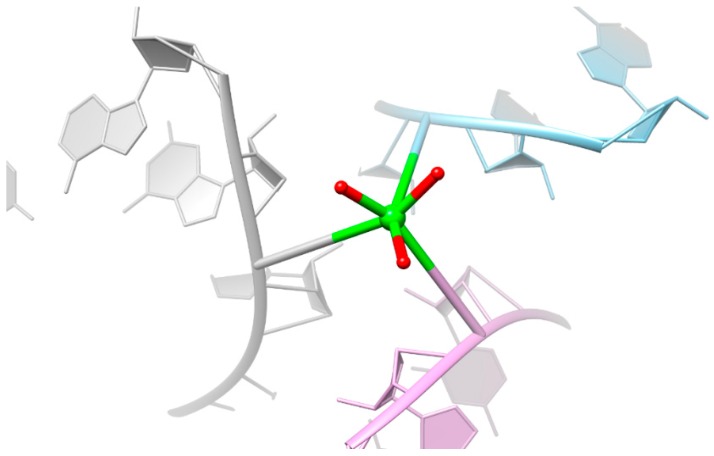
Representative illustration of Ca^2+^ (green sphere) connecting the three asymmetric units (in grey, light blue, and pink) by phosphate coordination. Coordinated water molecules are shown in red.

In every solved octameric structure one cation is located in the center of the major groove (Figure 2). The other metal ion is bound in an innersphere arrangement to the phosphate oxygen of guanine and further linked to neighboring asymmetric units in the crystal (Figure 3). We assume that the metal ion in the central part of the major groove of the duplex and the one sitting at the phosphate backbone have an effect on the compaction of the octameric RNA by compensating for the negative charge, which is generated through the approach of the two phosphate backbones in the center. In the RNA structure that was soaked in copper(II), the Ca^2+^ at the phosphate backbone is not replaced by Cu^2+^, which demonstrates the strong preference of Ca^2+^ for the phosphate oxygens.

Figure 2 shows that Tb^3+^ and Mn^2+^ occupy slightly different sites in the center of the RNA. Tb^3+^ is located towards the phosphate backbone and Mn^2+^ is closer to guanine, a preference which is observed in other macromolecules too [10,31]. There are additional ion binding sites in the presence of Mn^2+^, Co^2+^, and Cu^2+^.

### 2.3. Comparison of Observed Metal Ion RNA Interaction to Other Macromolecules as Suggested by the MINAS Database

**Figure 4:**
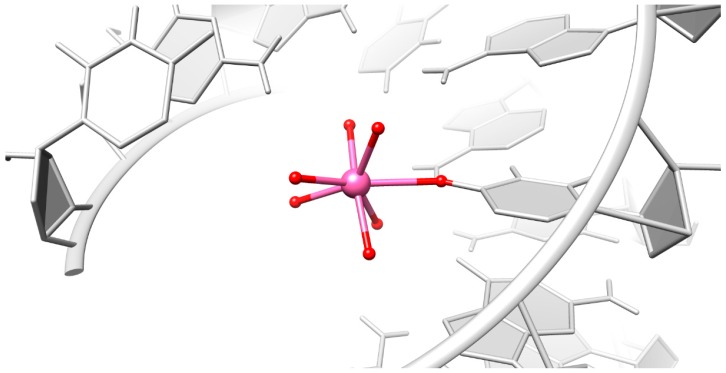
The unique innersphere interaction of Co^2+^ (pink sphere) to O4 of uracil with six additional coordinated water molecules (red spheres).

In addition to the innersphere binding to the phosphate oxygen of guanine, there is an uncommon innersphere binding to O4 of uracil by Ca^2+^, Co^2+^, and Cu^2+^ (Figure 4 and Table 2). While inner-sphere binding (e.g., to O6 of guanine [32]) is known, to the best of our knowledge innersphere binding to uracil O4 is here observed for the first time with Cu^2+^, compared to other macromolecules deposited in the protein databank (PDB) [33]. Outersphere binding to O6 of guanine is observed for all tested cations, except for Tb^3+^. In other macromolecules this interaction is frequently found for Mn^2+^ and Co^2+^, as suggested by the MINAS database [10]. Our study also confirms the preference of Tb^3+^, Ca^2+^, and Sr^2+^ to phosphate oxygens, as well as Co^2+^ and Cu^2+^ coordination to N7 of guanine [10,31], although in an outer-sphere manner in this octameric RNA duplex (Table 2).

### 2.4. Is There Cu^2+^ or Ca^2+^ in the Copper(II) Soaked Structure?

**Figure 5:**
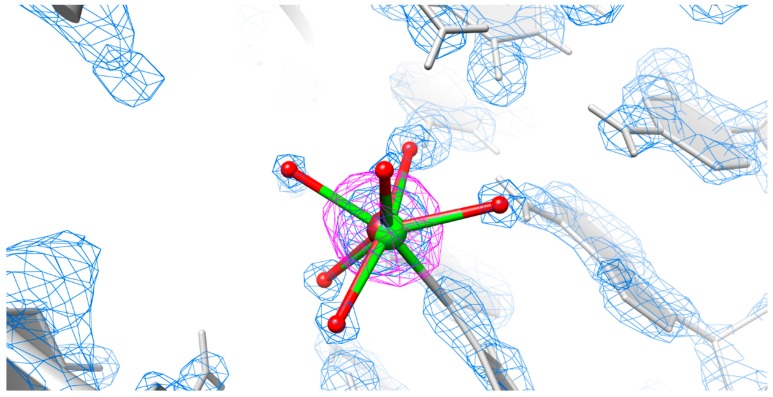
Representative model for the co-existence of Ca^2+^ and Cu^2+^. The RNA crystal structure demonstrates the mixed state of Ca^2+^ (green) and Cu^2+^ (brown) in the central part of the major groove. The anomalous difference map for Cu^2+^ shown as a pink mesh contours the atoms at the 4σ level and the electron density map shown as blue meshes contours the atoms at the 3σ level. Coordinated water molecules are shown as red spheres.

Although we observe a strong anomalous signal in the central part of the major groove at the absorption edge of Cu^2+^, the unusual elongated bond lengths to the coordinating waters [34,35] and the valence bond parameter [25] do not suggest that only Cu^2+^ is located at this position (Table 2). Therefore we try to quantitatively demonstrate the position of Cu^2+^ by integrating the area of the anomalous difference map. The occupancy of the strong anomalous scatterer Cu^2+^ relative to phosphorus, for which we assume 100% occupancy, was then calculated. The occupancy value for the anomalous difference map in Table 2 corresponds to the observed value from the electron density map and therefore confirms the position of Cu^2+^ at this site. The results indicate that the site is not fully occupied by Cu^2+^ and that in some structures Ca^2+^, instead of Cu^2+^, must be present (Figure 5).

## 3. Discussion

### 3.1. Structural Changes Are Independent of the Nature of the Cations

The more compact form of the solved RNA structures (Figure 1b and Table 2) clearly demonstrates the effects of di- and trivalent cations on RNA conformational changes, necessary for the catalytic activity in ribozymes or regulatory functions in riboswitches [36,37]. As we could not observe any conformational difference of the octameric RNA structures in the presence of different metal ions (Figure 1a), we assume that the specific position and orientation of the metal ions tend to be the determining factor for a fully active RNA rather than the nature of the metal ions. Hence, in RNA folding the choice of metal depends not only on physiochemical properties, but also on its abundance and availability. As *in vitro* evolution studies with the *Tetrahymena* group I and allosteric ribozymes in different metal ion environments demonstrate [38,39], RNA is even able to selectively adopt a metal ion.

### 3.2. Predominant Localization of All Tested Metal Ions in the Phosphate Backbone and in the Central Major Groove

The results propose that at least two metal ions are needed for structural changes and compensation of negative charges. The two negatively charged phosphate backbones approach each other at the major groove edge of the RNA duplex. This explains the localization of the metal ion at this specific position. Another preferred position is at the phosphate backbone, a position which is frequently reported in nucleic acids [10,40]. Different metal ions that occupy the same site in different crystal structures were already observed in previous studies [41,42,43,44]. Hence, crystallization buffers can also influence the metal ion binding sites and the occupancy, which should be considered when the position of the metal is related to functional activity of RNA.

Additional cations in the octameric RNA that are located at specific sites are Mn^2+^, Cu^2+^, and Co^2+^. They have smaller ionic radii compared to the other tested metal ions and are probably less sterically hindered to be in a closer position to the RNA duplex. Further, an additional Ca^2+^ is found at the phosphate backbone, reflecting the preference of the bigger-sized Ca^2+^ for this position [10].

Even if it is difficult to comment on the stabilities, as the resolution and the occupancy of the metal ion varies, we might observe a trend of “good” and “bad” binders, which corresponds to the Irving–Williams series [45,46]. Cu^2+^, Co^2+^, and Mn^2+^ seem to favor binding to the nucleobases, in contrast to Sr^2+^ or Ca^2+^ (Table 1). The dominant localization of Ca^2+^ at phosphate groups corresponds to reported increased stability constants for Ca^2+^ binding to the phosphodiester bridge [31].

Tb^3+^ and Mn^2+^ do not occupy the same central position observed for the other cations. As expected from other studies, Tb^3+^ is coordinated to phosphate oxygen atoms. Tb^3+^ competes with Mg^2+^ binding sites [47,48], hence the position of terbium(III) could suggest a possible Mg^2+^ binding site. Mg^2+^ is the most abundant metal ion in macromolecular structures [3,10]. However, we assume that Mg^2+^ is not required for the compact form of the octameric RNA structure. Mg^2+^ ions should be present in the case of the structure in the presence of Sr^2+^ and Co^2+^, but no specific structural change is seen compared to the other solved structures.

### 3.3. Particular Innersphere Binding of Co^2+^, Cu^2+^, and Ca^2+^ to O4 of Uracil

Innersphere binding to O4 of uracil is infrequent in other macromolecular structures, which is not surprising as uracil has basically no M^2+^ affinity, except if N3H is deprotonated [31,49]. There has been one case found for Co^2+^ in the crystal structure of the hammerhead ribozymes close to the cleavage site [50]. It was reported that Co^2+^ leads to higher *in vitro* cleavage rates than Mg^2+^ [51], however, there is no evidence that the localization of the Co^2+^ to this uracil O4 is the decisive factor.

Unexpectedly the coordination number of the central metal ion that undergoes this innersphere binding is seven, instead of six. However, transition metal ions are often flexible in both coordination number and geometry [52]. We assume that the compact conformation of the RNA squeezes the ligands and the surrounding water to close space in such a way that the correct geometry cannot be maintained. The compact conformation is either induced by the cations themselves, which counteract the negative charge, or it could be that the crystal packaging effects lead to structural changes in the RNA.

### 3.4. Mixed State Explains the Elongated Bond Length for the Copper(II) Soaked Crystal Structure

We found in the octameric RNA duplex a unique innersphere interaction of Cu^2+^ to O4 of uracil. Although copper is the third most abundant transition metal in the body and in the brain [9], interactions of copper(II) with nucleic acids are unusual [10], and to the best of our knowledge no RNA structure associated with copper has been deposited in the PDB [33].

The anomalous difference map clearly indicates the presence of copper(II) at this position. This was quantitatively demonstrated by integrating the anomalous difference map and by determining the occupancy of Cu^2+^ relative to the phosphates. However, the quantification of the anomalous signal also indicates that the position is not completely occupied by Cu^2+^. Hence, we propose a mixed state of Ca^2+^ with Cu^2+^ which was used for soaking (Figure 5).

## 4. Methods

### 4.1. RNA Synthesis and Purification

Chemically synthesized and desalted RNA oligonucleotides with the sequence 5′-UCGUACGA were purchased from Microsynth, Switzerland in a quantity of 2 μmol and purified by denaturing 20% polyacrylamide gel electrophoresis (PAGE) following standard procedures [53]. The excised gel slices were crushed and soaked twice in 10 mM Tris-HCl (pH 7.5) and 200 mM NaCl, 1 mM EDTA (pH 8.5). The supernatant was collected and precipitated by the addition of 100% EtOH and 50 mM NaCl. The RNA was desalted and concentrated to 1.2 mM.

### 4.2. RNA Crystallization and Soaking

RNA (0.6 mM) was annealed for 1 min at 50 °C in water and cooled to room temperature. Crystals were grown by the hanging drop vapor diffusion method in a 1:1 mixture of RNA and reservoir solution. The colorless needle-shaped crystals in the presence of Ca^2+^ grew after two days in a solution consisting of 200 mM CaCl_2_, 28% PEG 400, 2 mM spermine, and 50 mM hepes sodium buffer (pH 7.5). Soaking was done in reservoir solutions with 100 mM MnCl_2_, 2 mM TbCl_3_, or 20 mM CuCl_2_, instead of CaCl_2_, for 24 h. The conditions for the two co-crystallized RNA were 2 mM CoCl_2_, 20 mM MgSO_4_, 25% MPD, 0.5 mM spermine, and 50 mM potassium cacodylate (pH 6.0) (for the Co^2+^ crystals) and 200 mM SrCl_2_, 200 mM ammonium acetate, 10 mM magnesium acetate, 28% polyethylene glycol (PEG) 8000, and 50 mM sodium cacodylate pH 6.5 (for the Sr^2+^ crystals). Crystals were looped in cryosolution containing reservoir solution with 25% glycerol and flash-frozen in liquid nitrogen.

### 4.3. Data Collection and Structure Determination

Single crystal datasets were collected by beamline X06DA Swiss Light Source (Paul Scherrer Institute, Villigen, Switzerland) at 100 K with a PILATUS 2M-F detector (Dectris Ltd., Baden-Daettwil, Switzerland). Data were processed with XDS [54] and scaled with AIMLESS [55,56]. Highly redundant fine-φ sliced data [57] were collected at a single-wavelength with a multi-axis goniometer PRIGo (Table 3). The crystal in the presence of Ca^2+^ was solved with native-SAD at a wavelength of 1.61 Å (List 1). The long needle-shaped crystals were well-suited for measuring at different positions to prevent radiation damage and to get highly redundant data of high quality. All the other crystal structures were solved by molecular replacement using MOLREP [55]. Refinement cycles were performed in PHENIX [58] and molecular graphics and analyses were performed with the UCSF Chimera package [59]. Further details concerning data collection, phasing, and refinement are reported in Table 3 and List 1.

The ideal A-RNA duplex was predicted with the web-accessible tool RNAComposer [28].

**List 1.** Statistics of sub-structure determination and phasing (Ca^2+^ data).
SHELXD CC_weak_ (%)/CC_all_ (%) (for the top solution)48.8/29.3SHELXD CFOM (for the top solution)78.1SHELXD PATFOM (for the top solution)18.6Number of correct sites (for the top solution)14SHELXE CC (%)69.20SHELXE FOM0.656PHASER EP FOM0.697Map CC (%) (DM map against the map calculated from the refined model)82.4

### 4.4. Localization of Metal Ions

The identity of metal ions with sufficient anomalous scattering was confirmed by calculating an anomalous difference map in various resolution ranges with PHENIX [58]. The M–O distances to water were compared to data reported by Shannon [34,35]. The cutoff values for innersphere binding were set to 2.5 Å after the definition of the MINAS database [10], except for Sr^2+^, as the proposed M-O distance was 2.62 Å due to the longer ionic radii. For outersphere binding a maximum distance of 3.2 Å from water to RNA was set. The bond valence parameter as described in [25] is calculated for each observed metal-ligand distance R_i_ by *v_i_* = ∑exp((R_0_ − R_i_)/b), where R_0_ is a constant describing ideal distance, if the bond valence is 1, and b is an empirical constant. Both values are reported in [60]. The bond valence model was only employed for metal ion positions with an occupancy higher than 0.5.

For quantification of the anomalous signal of Cu^2+^ the anomalous difference map was integrated with Mapman [61] to add up the density inside a sphere of 2 Å around each atom. The f′′ value of Cu^2+^ and P were used to determine the ratio of their anomalous signals at the measured wavelength of 1.37 Å. The integrated value for phosphorus was then multiplied by this factor. For phosphorus, a 100% occupancy was assumed to calculate the relative occupancy for Cu^2+^.

## 5. Conclusions

Here we describe a detailed study of six different metal ions coordinating to a short RNA duplex. The cations have a rather strong influence on RNA structure, although none of the metal ions employed shows a specific individual structural influence. Different parameters were considered to carefully assign the positions of the individual metal ions, yielding two prevalent positions in the octameric RNA duplex. The first is coordinated to the phosphate backbone, the second cation binds in the major groove of the RNA, interacting by a particular innersphere coordination to O4 of uracil in the presence of Ca^2+^, Co^2+^, and Cu^2+^.

Finally, this study demonstrates the importance of considering several parameters when assigning metal ion positions in X-ray structures. Even when there is a strong anomalous signal present, we have to inspect other geometrical and crystallographic parameters for an accurate localization. On the other hand, geometrical consideration without examining the anomalous signal or other crystallographic parameters may lead to wrong interpretations as the geometry could be distorted by structural constraints of the RNA due to the metal ions or crystal packaging effects.

## Figures and Tables

**Table 1:** Selected helical parameters calculated with the program X3DNA [27]. The average values of all local base-pair values are provided together with the standard deviation as defined by the program X3DNA (in parentheses).

	Calculated 8mer Duplex	Ca^2+^	Mn^2+^	Co^2+^	Cu^2+^	Sr^2+^	Tb^3+^
Helical rise (Å)	2.80 (0.03)	2.4 (0.57)	2.59 (0.47)	2.41 (0.55)	2.43 (0.54)	2.58 (0.36)	2.56 (0.42)
Helical twist (°)	16.0 (0.9)	36.0 (2.7)	35.0 (2.4)	35.6 (2.9)	35.8 (3.1)	35.3 (1.8)	35.7 (1.7)
Major groove width (Å)	12.7	5.4	7.9	5.5	5.6	7.7	6.3

**Table 2:** Coordination of metal ions in the octameric RNA and comparison of observed RNA metal ion interactions to frequency reported in the MINAS database (*M*etal *I*ons in *N*ucleic *A*cid*S*) [10].

Metal ID	Cation	Inner Sphere Ligand ^a^	Distance (Å)	Occupancy	B-Factor	Outer Sphere Ligand ^b^	Valence ^c^	% in MINAS
1Ca/C1	Ca^2+^			1.0	19.4		2.2	
		O4 4U/A	2.39	1.0	18.1			0.7
		O 2HOH/D	2.43	1.0	21.7	N7 3G/A		3.9
		O 3HOH/D	2.45	1.0	21.5			
		O 14HOH/D	2.39	1.0	21.5	O6 3G/A		2.9
		O 15HOH/D	2.36	1.0	23.6			
		O 16HOH/D	2.35	1.0	18.4	O6 3G/B		2.9
		O 17HOH/D	2.4	1.0	18.1			
1Ca/E1	Ca^2+^			0.4	21.7			
		O2′/5 A/B	2.86	1.0	22.6			2.1
		O2′/5 A/B ^d^	2.86	1.0	22.6			2.1
		O2′/5 A/B ^d^	2.86	1.0	22.6			2.1
1Ca/F1	Ca^2+^			1.0	18.3		1.8	
		OP1 7G/A	2.34	1.0	21.0			18.7
		OP1 7G/A ^d^	2.34	1.0	21.0			18.7
		OP1 7G/A ^d^	2.34	1.0	21.0			18.7
		O 4HOH/D	2.52	1.0	26.1			
		O 4HOH/D ^d^	2.52	1.0	26.1			
		O 4HOH/D ^d^	2.52	1.0	26.1			
1Cu/C1	Cu^2+^			0.4 0.46 ^e^	19.1			
		O4 4U/A	2.40	1.0	18.1			0.0 (Cu^2+^) 0.7 (Ca^2+^)
		O 18HOH/D	2.32	1.0	19.1	O6 3G/A		10.3 (Cu^2+^) 2.9 (Ca^2+^)
		O 22HOH/D	2.30	1.0	18.3	O6 3G/A		10.3 (Cu^2+^) 2.9 (Ca^2+^)
		O 7HOH/D	2.38	1.0	22.9			
		O 5HOH/D	2.38	1.0	20.9	N7 3G/A		4.8 (Cu^2+^) 3.9 (Ca^2+^)
		O 2HOH/D	2.31	1.0	21.0	N4 2C/B		9.6 (Cu^2+^) 0.5 (Ca^2+^)
		O 4HOH/D	2.44	1.0	19.5			
1Ca/F1	Ca^2+^			0.2	19.1			
1Ca/G1	Ca^2+^			0.6	13.1		2.0	
		OP1 7G/A	2.35	1.0	15.4			18.7
		OP1 7G/A ^d^	2.35	1.0	15.4			18.7
		OP1 7G/A ^d^	2.35	1.0	15.4			18.7
		O 41HOH/D	2.39	1.0	19.1			
		41HOH/D ^d^	2.39	1.0	19.1			
		41HOH/D ^d^	2.39	1.0	19.1			
1Cu/C4	Cu^2+^			0.2	25.1		-	
		O 49HOH/D	2.19	1.0	30.6	N7 8A/B		1.4
		O 47HOH/D	2.22	1.0	34.2	N6 8A/B		1.2
		O 85HOH/D	2.16	0.5	33.1			
		O 86HOH/D	2.15	0.4	32.1			
		O 1HOH/E	2.21	1.0	36.1			
		O 70HOH/D ^d^	2.11	1.00	36.6			
1Co/E1	Co^2+^			0.5	8.5		2.1	
		O4 4U/A	2.42	1.0	14.4			3.9
		O 1HOH/C	2.13	1.0	12.8	O6 3G/B		11.1
		O 13HOH/C	2.11	1.0	15.9	O6 3G/A		11.1
		O 4HOH/C	2.10	1.0	15.5			
		O 6HOH/C	2.11	1.0	20.6	N7 3G/A		10.2
		O 5HOH/C	2.13	1.0	17.5			
		O 3HOH/C	2.10	1.0	20.4			
1Co/D2	Co^2+^			0.6	12.0		1.8	
		O 1HOH/C	2.11	1.0	18.9			
		O 1HOH/C ^d^	2.11	1.0	18.9			
		O 1HOH/C ^d^	2.11	1.0	18.9			
		OP1 7G/A	2.15	1.0	15.3			2.6
		OP1 7G/A ^d^	2.15	1.0	15.3			2.6
		OP1 7G/A ^d^	2.15	1.0	15.3			2.6
1Co/E2	Co^2+^			0.3	12.5			
		O 81HOH/C	2.10	1.0	23.7	N7 7G/A		10.2
		O 37HOH/C	2.07	1.0	25.3			
		O 17HOH/C	2.12	1.0	20.3	OP2 6C/A		5.1
		O 61HOH/C	2.11	1.0	26.0			
		O 63HOH/C	2.10	1.0	27.6			
		O 62HOH/C	2.09	1.0	32.4			
1Mn/C1	Mn^2+^			0.7	14.7		2.1	
		O 1HOH/F	2.14	1.0	20.4			
		O 2HOH/F	2.18	1.0	19.5	O4 4U/A		3.3
		O 3HOH/F	2.08	1.0	16.5	O6 3G/B		9.3
		O 15HOH/F	2.19	1.0	21.4	N7 3G/B		8.0
		O 16HOH/F	2.31	1.0	18.4			
		O 17HOH/F	2.21	1.0	22.9			
1Mn/D1	Mn^2+^			0.6	14.0		2.5	
		O 7HOH/F	2.17	1.0	18.9	O6 7G/A		9.3
		O 8HOH/F	2.21	1.0	23.2			
		O 9HOH/F	2.00	1.0	22.2			
		O 10HOH/F	2.08	1.0	25.5			
		O 11HOH/F	2.05	1.0	23.4			
		O 12HOH/F	2.18	1.0	18.9	N7 7G/A		8.0
1Mn/E1	Mn^2+^			0.4	15.3			
		O 13HOH/F	2.10	1.0	25.9			
		O 14HOH/F	2.07	1.0	23.1	O6 7G/B		9.3
		O 44HOH/F	2.13	1.0	26.2	N7 7G/B		8.0
		O 45HOH/F	2.06	1.0	30.6			
		O 37HOH/F ^d^	2.32	1.0	26.5			
		O 43HOH/F ^d^	2.23	1.0	26.2			
1Mn/G1	Mn^2+^			0.7	22.2			
		OP1 7G/A	2.59	1.0	28.4			4.6
		OP1 7G/A ^d^	2.99	1.0	28.4			4.6
		OP1 7G/A ^d^	2.27	1.0	28.4			4.6
1Sr/C2	Sr^2+^			0.6	28.5		1.4	
		O 1HOH/N	2.52	1.0	33.6	O4 4U/A		5.4
		O 2HOH/Q	2.61	1.0	42.5			
		O 3HOH/D	2.49	1.0	36.3	O6 3G/B		5.2
		O 4HOH/D	2.53	1.0	30.3	O6 3G/A		5.2
		O 6HOH/D	2.61	1.0	35.92			
		O 7HOH/D	2.62	1.0	38.4			
1Sr/F1	Sr^2+^			0.5	35.3			
		OP1 7G/A	3.20	1.0	33.9			
		OP1 7G/A ^d^	3.20	1.0	33.9			
		OP1 7G/A ^d^	3.20	1.0	33.9			
1Tb/1	Tb^3+^			0.4	39.4			
		OP2 2C/A	2.53	1.0	40.9			-
		OP2 2C/B	2.50	1.0	41.6			-
		O 1HOH/	2.34	1.0	45.0			
		O 14HOH/D	2.30	1.0	45.1			
		O 19HOH/D	2.55	1.0	46.6	O5′/1 U/A		7.1
1Tb/E2	Tb^3+^			0.2	44.4			
		OP1 7G/A		1.0	41.8			-
		OP1 7G/A ^d^		1.0	41.8			
		OP1 7G/A ^d^		1.0	41.8			

^a^ The cutoff values for innersphere binding were set to 2.5 Å, as defined in the MINAS database. For Sr^2+^ the cutoff value was set to 2.62 Å according the theoretically expected value; ^b^ after the definition of the MINAS database a maximum distance of 3.2 Å from H_2_O to RNA was set; ^c^ for atoms with low occupancy the valence bond parameter was not calculated; ^d^ symmetry-related atoms; ^e^ occupancy calculated from the anomalous signal.

**Table 3:** Statistics of data collection and refinement of the octamer in the presence of the indicated divalent metal ions.

	Ca^2+^	Mn^2+^	Co^2+^	Cu^2+^	Sr^2+^	Tb^3+^
PDB Code	4U3L	4U3O	4U3R	4U78	4U3P	4U47
Data collection						
λ (Å)	1.60810	1.60000	1.60000	1.37478	1.60000	1.60000
Exposure period (s)	0.1	0.1	0.1	0.1	0.1	0.1
Oscillation range (°)	0.25	0.1	0.1	0.1	0.1	0.1
Space group	H3	H3	H3	H3	H3	H3
Unit cell parameters						
a (Å)	46.88	46.33	46.97	46.81	45.48	46.51
b (Å)	46.88	46.33	46.97	46.81	45.48	46.51
c (Å)	53.15	58.08	53.39	53.38	57.98	56.63
Resolution range (Å)	32.00–1.68	23.16–1.8	32.34–1.72	32.28–1.50	32.58–1.87	32.82–1.95
Number of reflections						
Total	69,346	78,548	35,809	112,323	35,169	31,751
Unique	4726	4140	4265	6864	3728	3225
Completeness (%) ^a^	95.2 (77.60)	95.74 (79.91)	96.75 (84.68)	98.39 (90.32)	99.79 (97.96)	96.82 (89.57)
(*I*)/(*σ*(*I*)) ^a^	58.00 (16.2)	43.26 (12.06)	14.60 (12.22)	48.32 (4.56)	34.84 (4.06)	33.19 (5.35)
Average multiplicity	14.7(4.9)	19.0(11.4)	8.4 (2.0)	16.4 (5.0)	9.4 (7.0)	9.8 (9.0)
R_meas_^a^	0.036 (0.076)	0.059 (0.23)	0.042 (0.044)	0.041 (0.042)	0.037 (0.48)	0.052 (0.44)
CC1/2	100 (97.7)	100 (99.2)	99.8 (82.4)	99.9 (98.2)	99.9 (98.2)	99.8 (98.6)
Refinement						
R_work_	0.187	0.165	0.165	0.177	0.180	0.172
R_free_	0.212	0.175	0.198	0.206	0.229	0.207
Root mean square deviations (r.m.s.d.) from target values						
Bond lengths (Å)	0.005	0.005	0.002	0.002	0.004	0.004
Bond angle (Å)	0.890	0.910	0.400	0.400	0.740	0.780
Average B-factors (Å^2^)						
Ligands	24.5	16.6	10.9	20.3	32.4	41.8
RNA	22.9	21.4	15.1	18.1	28.7	39.0
Solvent	27.1	26.1	25.9	28.5	34.2	43.9
Number of RNA atoms ^b^	334	334	334	334	334	334
Number of solvent molecules	29	65	87	78	64	37

^a^ Statistics for the highest-resolution shell are shown in parentheses; ^b^ per asymmetric unit.

## Protein Data Bank Accession Codes

The atomic coordinates are available at the RCSB Protein Data Bank under the accession code 4U3L, 4U3R, 4U78, 4U3O, 4U3P, and 4U47.
